# Editorial: Sedentary behavior and health outcomes. Origins, mechanisms, and policy

**DOI:** 10.3389/fphys.2023.1197926

**Published:** 2023-04-13

**Authors:** Erik D. Hanson, Simon Fryer, Simon Higgins, Lee Stoner

**Affiliations:** ^1^ Department of Exercise and Sport Science, University of North Carolina, Chapel Hill, NC, United States; ^2^ Lineberger Comprehensive Cancer Center, University of North Carolina, Chapel Hill, NC, United States; ^3^ School of Natural, Social and Sport Sciences, University of Gloucestershire, Gloucester, Gloucestershire, United Kingdom; ^4^ Department of Epidemiology, Gillings School of Global Public Health, University of North Carolina, Chapel Hill, NC, United States; ^5^ Center for Health Promotion and Disease Prevention, University of North Carolina, Chapel Hill, NC, United States

**Keywords:** sedentary behaviior, physical inactivity, cardiometabolic health, prolonged sitting, cardiovascualr disease

## 1 Introduction

Sedentary behaviors (SB), defined as low-intensity activities in a seated, reclined, or supine positions, have become a ubiquitous aspect of contemporary societies. Accordingly, international public health authorities, such as the World Health Organization, now recognize the importance of SB as it relates to health outcomes, including but not limited to: cardiometabolic diseases, some cancers, quality of life, and mental health. As highlighted by Higgins et al., SB research is in its infancy and there is much work to be done before we as a research community can inform policy beyond the vague advice of “move more and sit less”.

Population-level increases in SB coupled with decreasing levels of physical activity (PA) have negative implications on cardiovascular disease (CVD) rates, which is the leading cause of death world-wide. CVD risk is multi-factorial and is influenced by many different lifestyle factors highlighted in this Research Topic, such as poor diet (Fryer et al.), reduced PA (Vaara et al.; Bowden Davies et al.), and increased sitting time (Bowden Davies et al.; Zimmer et al.). Lifestyle behaviors are complex and interrelated, with individual-, social-, and environmental-level determinants, but are also modifiable with improved diets and greater activity levels working to reduce low-grade inflammation that hastens chronic disease progression (Huston). However, by examining the origins of SB, we can begin to understand how these occurrences came to be and are better positioned to develop strategies to sustainably change behavior and inform policy (Higgins et al.).

This Research Topic examined SB in the context of its evolution, biological plausibility for associations with health outcomes, interruption interventions, and implementation through to policy. It includes 6 papers (Vaara et al.; Bowden Davies et al.; Higgins et al.; Huston; Zimmer et al.; Fryer et al.). Within this Research Topic, cardiometabolic health outcomes ranged widely, but were consistently investigated within the context of SB and/or PA.

## 2 Health outcomes

Examining SB and health outcomes was a most common approach within this Research Topic. Previously, self-reported PA and SB have been linked with cardiorespiratory fitness, but such measures lack objectivity and can lead to over/under-reporting. Here, objective tracking devices were used to improve outcome reporting accuracy, with PA and SB being positively and negatively associated with cardiorespiratory fitness, respectively (Vaara et al.). Moreover, PA and SB were also associated with lower body fitness (standing long jump, maximal isometric force) in a similar manner, extending knowledge in this area. Next, 14 days of step reduction in habitually active individuals (>10,000 steps/day) decreased endothelial function (flow mediated dilation), insulin sensitivity, and cardiorespiratory fitness while increasing total body fat (Bowden Davies et al.). However, these impairments were all reversed upon resumption of normal activity levels. Finally, poor dietary choices and prolonged SB are often co-occurring behaviors. Meals with high fat content are a potentially important factor associated with increased CVD prevalence. Supporting this idea, an acute bout of 180 min of sitting coupled with a low-fat meal revealed negligible changes in carotid-femoral pulse wave velocity, though prolonged sitting coupled with a high fat meal led to significantly increased (worsened) arterial stiffness (Fryer et al.). Importantly, heel raises performed every 5 min abrogated the adverse effects of the high fat meal. Together, transitory periods of decreased PA that consequently increased SB led to acute negative changes in markers of cardiovascular health that can be reversed with resumption of normal activity.

## 3 SB interruption interventions

Effective SB interruption strategies was the second major theme, although this frequently intertwined with Health Outcomes as both Bowden Davies *et al.* (Bowden Davies et al.) and Fryer et al. incorporated interruption strategies to improve their targeted health outcomes. In addition, the context of SB is a growing area of investigation, with the impact of different types of SB on cardiometabolic outcomes receiving recent attention. However, amateur e-sports report no changes in energy expenditure, respiratory or cardiovascular parameters during 30 min of videogame play (Zimmer et al.), suggesting that whilst short bouts of video gaming may not cause acute cardiovascular dysfunction, they also do not provide the benefits of traditional PA and likely contribute to increased SB time. Collectively, regular muscle contractions appear to be a necessary factor leading to healthy endothelial function and maintaining arterial stiffness. However, identifying ways to integrate greater movement into current lifestyles along through the identification of barriers and facilitators SB along with practical disruption doses remain to be determined.

## 4 Sedentary behavior evolution and context

The remaining papers provided an evolutionary perspective and a network analysis. Higgins and others (Higgins et al.) discuss how ancestral SB and PA likely differed from the modern world, including co-occurring behaviors (e.g., eating, screen time, mental stress) that may accelerate CVD risk. The authors highlight that the scientific literature has a long-standing emphasis on achieving adequate PA levels, yet ironically many of these same active individuals also engage in high volumes of SB. Continued efforts that improve awareness of SB are essential, with development of specific guidelines that are on par with sleep (7–9 h per night) and PA (>150 min/week) being a key towards policy changes. A network analysis provides a theory for how unhealthy lifestyle factors (high SB/low PA) lead to chronic disease (Huston). These included (i) the macroenvironment, (ii) social/culture factors, (iii) lifestyle, (iv) physiological systems, (v) the microenvironment and (vi) intracellular networks. Low-grade, sustained inflammation is argued to be a principal underlying factor within the body, stemming from poor diet, along with socioeconomic and education disparities that contribute to reduced PA and increased SB which are exacerbated by global instability.

## 5 Conclusion

This Research Topic has helped address some of the pressing research questions related to SB in the context of its evolution, associations with health outcomes, and potential interruption strategies. While the goal of implementation of specific policies to reduce SB remains unfulfilled, studies of these nature are necessary first steps. As the world emerges from the COVID-19 pandemic, there is an opportunity to progress development of guidelines that ultimately can be used to inform policy. From a practical standpoint, effective, evidence-based tools to sustainably reduce SB are needed to assist practitioners and policymakers in the development of population specific health education plans and reduce the increasing prevalence and severity of non-communicable diseases.

